# 

**Published:** 1872-05

**Authors:** 


### TABLE OF CONTENTS.

ORIGINAL COMMUNICATIONS:

REPORTS OF SURGICAL CASES. By F. A. STANFORD, M.D., Columbus, Ga.. 65
(Placenta Prævia—Treatment by Rupture of the Membrane, Evacuation of the Liquor Amnii, and Separation of the Placenta from its Uterine Connections.——Puerile Incontinence of Urine—Treatment by Bougie.——A Hydatid Mass Taken from the Uterus.——Hydatid Tumor, Involving the Deltoid Muscle, from which Twenty-one Distinct Cysts were Removed.)

ABSTRACTS FROM GERMAN JOURNALS. By CH. RAUSHENBERG, Atlanta, Ga., 72
(On the Treatment of Varix by Subcutaneous Injections of Ergotine. By Dr. Paul Vogt, Assistant Physician to the Surgical Polyclinic at the University of Greifswalde.——Apomorphine—A New Remedy.)

CYSTIC AND FATTY TUMOR OF THE RECTUM. Reported by W. D. HOYT, M.D., Rome, Georgia .......... 78

HYDRATE OF CHLORAL. By C. P. WHITEHEAD, M.D., Lake Providence, La. 80

ANNUAL ADDRESS. Delivered Before the Georgia Medical Association, at its Twenty-Third Session, Held in Columbus, Georgia, April 10, 1872. By the President, GEO. M. McDOWELL, M.D., Barnesville, Georgia.... 82

WELCOME ADDRESS. Delivered to the Georgia Medical Association, April 10, 1872. By V. H. TALIAFERRO, M.D., Columbus, Georgia ........ 92

THE RELATION BETWEEN THE PHYSICIAN AND PHARMACIST. By TH. SCHUMANN, Atlanta, Georgia.......... 96

REPORTS OF CASES. By CARLISLE TERRY, M.D., Columbus, Georgia. ..... 100
(Gun-Shot Wounds of the Brain.—Case of Superfœtation.)

BROMIDE OF POTASSIUM. By K. P. MOORE, M.D., Knoxville, Georgia .... 103

FOREIGN CORRESPONDENCE:

NEW REMEDIES IN THE TREATMENT OF UNUNITED FRACTURES. By A. W. CALHOUN, M.D., Berlin, Prussia.......... 108

EDITORIAL AND MISCELLANEOUS:

EDITORIAL CORRESPONDENCE.......... 113

CIRCULAR.......... 115

BROMIDE OF POTASSIUM IN EPILEPSY.......... 115

ADVERTISING SHEET.......... 115

BIBLIOGRAPHICAL.......... 116

